# Patterns of complementary and alternative medicine use and factors influencing its utilization in thyroid cancer: findings from the MASTER study

**DOI:** 10.1186/s12906-026-05428-w

**Published:** 2026-06-23

**Authors:** Han-Sang Baek, Mijin Kim, Kyungsik Kim, Woojin Lim, Kwangsoon Kim, Ja Seong Bae, Dong-Jun Lim, Eun-Jae Chung, Bon Seok Koo, Sung Yong Choi, Eun Kyung Lee, Eu Jeong Ku, Kyeong Jin Kim, Hee Kyung Kim, June Young Choi, Bo Hyun Kim, Sue K. Park

**Affiliations:** 1 Division of Endocrinology and Metabolism, Department of Internal Medicine, College of Medicine, Uijeongbu St. Mary’s Hospital, The Catholic University of Korea, Seoul, Republic of Korea; 2 Department of Internal Medicine, Biomedical Research Institute, Pusan National University Hospital, Pusan National University School of Medicine, Busan, Korea; 3 Department of Preventive Medicine, Seoul National University College of Medicine, 103 Daehak-ro, Jongro-gu, Seoul, 03080 Korea; 4 Cancer Research Institute, Seoul National University, Seoul, Korea; 5 Institute of Environmental Medicine, Medical Research Center, Seoul National University, Seoul, Korea; 6 Department of Surgery, College of Medicine, The Catholic University of Korea, Seoul, Republic of Korea; 7 Division of Endocrinology and Metabolism, Department of Internal Medicine, Seoul St. Mary’s Hospital, College of Medicine, The Catholic University of Korea, Seoul, Republic of Korea; 8 Department of Otorhinolaryngology-Head and Neck Surgery, Seoul National University Hospital, Seoul National University College of Medicine, Seoul, Korea; 9 Department of Otorhinolaryngology–Head and Neck Surgery, College of Medicine, Chungnam National University, Daejeon, Korea; 10 Department of Otorhinolaryngology-Head and Neck Surgery, National Cancer Center, Goyang, Korea; 11 Department of Internal Medicine, Center for Thyroid Cancer, National Cancer Center, Goyang, Korea; 12 Division of Endocrinology and Metabolism, Department of Internal Medicine, Seoul National University Hospital Healthcare System Gangnam Center, Seoul National University College of Medicine, Seoul, Korea; 13 Division of Endocrinology and Metabolism, Department of Internal Medicine, Korea University College of Medicine, Seoul, Korea; 14 Department of Internal Medicine, Chonnam National University Medical School, Gwangju, Korea; 15 Seoul National University Bundang Hospital, Seongnam-si, Gyeonggi-do Korea; 16 Integrated Major in Innovative Medical Science, Seoul National University Graduate School, Seoul, Korea

**Keywords:** Complementary and alternative medicine, Thyroid cancer, MASTER study

## Abstract

**Background:**

Complementary and alternative medicine (CAM) therapies are increasingly popular; however, comprehensive data on their usage patterns and types remain limited, especially in South Korea. We investigated the patterns and economic impact of CAM use in thyroid cancer after lobectomy.

**Methods:**

We utilized data from the MASTER study, a prospective, multicenter, randomized, controlled trial on patients with differentiated thyroid cancer post-lobectomy. Data on CAM usage in 1,166 patients at baseline and at 3, 6, and 12 months postoperatively were included. Logistic regression quantified the effect of levothyroxine treatment on CAM usage.

**Results:**

CAM use was reported by 58% of participants. Dietary nutritional products were the most used CAM modality, with usage rates of 42.5% at baseline, decreasing to 37.7% at 12 months. Exercise and physical therapies also showed significant differences, with 19.2% using these modalities at baseline, increasing to 27.1% at 12 months. The median cost for CAM varied, with dietary products consistently costing 144 USD (200,000 KRW) and exercise costs rising from 144 USD (200,000 KRW) to 288 USD (400,000 KRW) over time. Patients on levothyroxine were less likely to use CAM, with an odds ratio (OR) of 0.57 (95% confidence interval [CI] = 0.45–0.72, *P* < 0.001). However, levothyroxine users who did use CAM were more likely to use multiple CAM modalities, with an OR of 3.66 (95% CI = 1.88–7.14, *P* = 0.001) at baseline, though this association did not remain significant later.

**Conclusions:**

CAM is integrated into thyroid cancer treatment, indicating a need for healthcare professionals to discuss its safety and effective care integration.

**Supplementary Information:**

The online version contains supplementary material available at https://doi.org/10.1186/s12906-026-05428-w.

## Background

Complementary and Alternative Medicine (CAM) refers to a diverse group of medical practices and products not considered part of conventional medicine, ranging from herbal supplements and acupuncture to mind-body practices [1]. Alongside conventional medical treatment, there is a growing interest in the role of CAM in patients with cancer, driven by increasing patient desire for holistic care, perceived limitations of conventional therapies, and efforts to manage cancer-related symptoms and improve quality of life [2–4]. Although there were differences depending on the study, as many as 30% or as many as 50% or more of patients with cancer reported having used CAM [5–9].

Regarding thyroid cancer, one of the most common endocrine malignancies, the interest in CAM is similar to that in other cancers [10]. Patients with thyroid cancer often face unique challenges, including long-term hormone replacement therapy and monitoring, owing to the indolent features of thyroid cancer [11–14]. Despite excellent prognosis, thyroid cancer survivors report wide QoL impacts, especially after more extensive surgery—driving interest in supportive, holistic options [15]. In this regard, patients with thyroid cancer are more likely to be exposed to CAM use. In a study on patients with thyroid cancer conducted in the United States (US), 70% of study participants used CAM, which was reported to be about twice that of the general population [10]. Additionally, using CAM therapies can lead to increased direct and indirect healthcare expenditures, raising concerns about the economic implications for patients and healthcare systems. In a study from the US, adult cancer survivors spent an estimated annual 6.7 billion USD on vitamins or minerals and CAM. This accounted for over 11.4% of the annual out-of-pocket costs [16]. In one study from South Korea, of the 186 cancer patients hospitalized in one tertiary hospital, 78.5% used at least one type of CAM and spent approximately $ 1,100 USD monthly on CAM [17].

As the popularity of CAM therapies increases, the evidence base supporting CAM must also continue to grow [18, 19]. However, despite the apparent popularity of CAM among these patients and concern about increasing healthcare expenditures, there is a notable lack of comprehensive data on its usage patterns, types, and efficacy, particularly in South Korea [14, 20]. For instance, in a South Korean study on lymphoma survivors, 77.5% of CAM users did not consult doctors citing “no need” (66.5%), “doctor’s negative response” (17%), and “doctor’s lack of CAM knowledge” (10.0%) as the reasons [21]. Therefore, in this study, we aimed to shed light on the prevalence, types, and economic impact of CAM in the population of patients with thyroid cancer. In this study, we adopt the National Center for Complementary and Integrative Health (NCCIH) usage of terms and use ‘CAM’ as an umbrella term for non-mainstream practices used with (complementary) or instead of (alternative) conventional medicine [1]. In our analysis, CAM follows the survey categories used in this study, including pharmacotherapy, phytotherapy or nutritional supplementation, traditional Korean medicine and other oriental therapies, dietary nutritional products, mind–body interventions, physical therapies, and exercise.

## Materials and methods

### MASTER trial

This CAM study is a sub-group of the MASTER trial (Multicenter, Randomized, Controlled Trial for Assessing the Usefulness of Suppressing Thyroid Stimulating Hormone Target Levels after Thyroid Lobectomy in Low to Intermediate Risk Thyroid Cancer Patients) [22]. The MASTER trial, conducted across multiple centers, is a prospective, randomized, controlled trial aiming to assess the effectiveness of thyroid stimulating hormone (TSH) suppression in patients with papillary thyroid cancer (PTC) following lobectomy. Detailed eligibility criteria and study protocol are provided in the Supplementary Material I. Patients were recruited before lobectomy after diagnosis of thyroid cancer. Those who agreed to participate in the trial were randomized into the low-TSH group (target TSH 0.3 ~ 1.99 µIU/mL) or high-TSH (target TSH 2.0 ~ 7.99 µIU/mL) group, and levothyroxine (LT4) was administered as needed to reach a target range. The 2015 ATA guidelines recommend TSH suppression for high-risk thyroid cancer and a weak recommendation for TSH levels of 0.5–2.0 uIU/mL for lower-risk cases; MASTER study seeks reliable data on TSH target levels, setting the upper normal at 8.0 mIU/L based on Korean health survey data [23, 24]. After randomization, patients were requested to visit an outpatient clinic every 3 months during the first 6 months, every 6 months for 2 years, and annually thereafter. The study protocol was reviewed and approved by the Institutional Review Board of each participating institution (trial registration number: KCT0004989). This trial was registered at the Clinical Research Information Service (CRIS) on May 6, 2020 (https://cris.nih.go.kr/cris/search/search_result_st01.jsp?seq=16437). The written informed consent was received from all study participants.

### Data collection for CAM

The survey was conducted to examine the cost and usage pattern of CAM in patients with thyroid cancer. A questionnaire on CAM was collected to assess medical costs, and the survey was conducted at the time of enrollment preoperatively (baseline) and 3, 6, and 12 months from the enrollment. A CAM survey was not conducted at 24-month. Because the CAM questionnaire was not mandatory in the MASTER study, some patients did not participate in the survey. Between May 2020 and September 5, 2023, a total of 2,407 patients were enrolled in the MASTER study. Among them, 1,166 patients (48.4%) completed the CAM survey and were included in this analysis. The remaining 1,241 patients (51.6%) did not respond to the CAM survey and were therefore excluded. To assess potential selection bias, a comparative analysis between responders and non-responders was conducted, and the results are presented in Supplementary Table 1.

Patients who responded “yes” to using CAM at least once at the survey of baseline, 3-month, 6-month and 12-month from the enrollment in the MASTER trial were classified to CAM users (*N* = 676). The number of CAM uses was calculated by checking how many types of CAM were used in 4 surveys from registration to 12-month follow-up (FU), and the total cumulative cost over 12 months was calculated. In addition, to determine whether there was a change in CAM use patterns and costs paid from baseline to 12 months, the types of CAM use and costs paid due to CAM for 1,166 patients were calculated separately for baseline, 3-month, 6-month, and 12-month FU. (Supplementary Fig. 1)

For example, if a subject did not use CAM at baseline, but used pharmacotherapy at 3 months, used nutritional supplements at 6 months, and then did not use CAM at 12 months, this was recoded as using both pharmacotherapy and nutritional supplements, the number of CAM use types was recoded into two, and the total cost spent on CAM for the person was calculated by adding the 3-month cost and the 6-month cost. In the calculation at each follow-up time, it was calculated based on whether each CAM modality was used at that FU time, the number of CAM use types, and the cost paid.

### Questionnaire of CAM

The questionnaire included information on whether CAM was used and the costs paid in the following eight items: (i) pharmacotherapy (*prescribed by a physician)*, (ii) phytotherapy or nutritional supplementation (*purchased from a pharmacy)*, (iii) traditional Korean medicine therapies (*prescribed by a Korean medicine doctor)*, (iv) other traditional oriental therapies (Korean folk medicines, i.e. bee acupuncture, moxibustion, cupping, etc.), (v) dietary nutritional products (such as health aids, fermented liquids), (vi) mind-body interventions (i.e. prayer, Buddihist chants, etc.), (vii) Physical therapies (such as hot springs, massages, etc.), (viii) exercise (i.e. fitness training, yoga, etc.). For each area, participants were asked if they used the CAM in the past 3 months, with the option to select multiple therapies. For each CAM modality, participants were asked to indicate whether they had used the modality (yes/no) and, if applicable, to report the corresponding out-of-pocket cost. Participants were also asked to provide brief descriptions of the specific CAM modalities used. Specifically, the expenditure component of the CAM questionnaire was developed with reference to the healthcare expenditure items of the 2018 Korea Health Panel Survey (KHP), a nationally representative survey of healthcare utilization and medical spending [25]. However, rather than using predefined expenditure categories, participants were asked to directly report out-of-pocket costs for each CAM modality. The full CAM questionnaire is provided as Supplementary Material II.

None of 8 CAM modalities are covered by the National Health Insurance Service or other private health insurance in Korea. The survey was conducted in a self-administered questionnaire. However, to improve the completeness of the survey, a confirmation process by the nurse research coordinator was included to ensure that the patient understood the questionnaire well and to review any missing information before completion of survey. The survey was conducted for cost investigation in patients with thyroid cancer. Owing to the study design, we did not examine whether the participants disclosed their using CAM modalities to their thyroid cancer clinicians. The questionnaire was developed based on the MASTER study framework and was reviewed by clinical experts in endocrinology to ensure content validity, including assessment of item relevance and clarity. However, no pilot or feasibility testing or independent psychometric validation was conducted, as the instrument was designed for exploratory assessment within the context of this study. The instrument was designed to capture behavioral aspects of CAM use, including modality utilization and associated costs, rather than to function as a psychometric scale.

### Risk factors affecting CAM use

Demographic variables such as age, gender, occupation, etc., tobacco smoking and alcohol drinking habits and amount, family history, and personal disease history were surveyed through questionnaires at the time of enrollment in the MASTER trial. Height and weight measurements were taken at each visit, and the baseline BMI used in the analysis was calculated from measurements taken within a month during the periods before lobectomy and randomization. For the analysis of risk factors affecting CAM use, BMI at the baseline was included in the multiple logistic regression model. The randomization group and LT4 use were confirmed in the medical record at the time of enrollment. LT4 doses were identified in the medical record at the time of 3-month of follow-up after randomization. The demographic variables were those examined at the baseline. The risk factors affecting CAM use were those examined at the baseline.

Comorbidities were identified through patient surveys and included the following conditions: diabetes mellitus, hypertension, hyperlipidemia, heart/brain diseases, osteoporosis, hyperthyroidism, asthma/chronic obstructive pulmonary disease, female hormone therapy, rheumatoid arthritis, type 1 diabetes, inflammatory bowel disease, fractures, cancers other than thyroid cancer, and others.

### Statistical analysis

We described continuous variables as means and standard deviations, and categorical variables as frequencies and percentages. The t*-*test was used to analyze continuous variables, whereas the chi-squared test was applied to categorical variables. Post-hoc analyses were performed to investigate the frequency of CAM use according to TSH levels and LT4 use, generating hypotheses about their potential associations. We evaluated the likelihood of CAM use with the odds ratios (ORs) and 95% confidence intervals (CIs) in multiple logistic regression model to identify factors affecting CAM use compared to CAM non-use. The multivariable model included current alcohol drinking, obesity, (≥ 25 kg/m^2^), LT4 use, and randomization group (High-TSH vs. Low-TSH groups), sex and age. The variables of ‘current alcohol drinking’, ‘obesity’, and ‘LT4 use’ were selected as covariates because differences in the distribution were observed between the CAM users and the non-users. The randomization group was also included as a covariate since the ‘LT4 use’ could be administered differently to reach different TSH target ranges in each randomization group in the MASTER trial. Statistical analyses were conducted using R software (version 3.6.2; R Foundation for Statistical Computing, Vienna, Austria; available at http://www.R-project.org).

## Results

### Characteristics of study population

Table 1 presents the characteristics of 1,166 patients with PTC divided according to CAM use. Among the total number of patients, 490 (42.0%) were non-CAM users, and 676 (58.0%) were CAM users. Among the CAM users, 337 (49.9%) reported using one type of CAM, and 339 (50.1%) reported using two or more types.

**Table 1:** Characteristics of PTC patients who underwent lobectomy according to the CAM use from thyroid cancer diagnosis to 12 months of follow-up in the MASTER trial

Characteristics at the baseline	Total PTC patients (*n* = 1,166)	CAM non-user^1^ (*n* = 490)	CAM user^1^ (*n* = 676)	*P*-value^2^
Random-assigned two arms				0.482
Low-TSH group	584 (50.1)	239 (48.8)	345 (51.0)	
High-TSH group	582 (49.9)	251 (51.2)	331 (49.0)	
Age (years old)				0.376
< 40	328 (28.1)	135 (27.6)	193 (28.6)	
40–59	631 (54.1)	259 (52.9)	372 (55.0)	
60	207 (17.8)	96 (19.6)	111 (16.4)	
Sex, male	263 (22.6)	120 (24.5)	143 (21.2)	0.203
BMI (kg/m^2^) ≥ 25 kg/m^2^ (Obesity)	511 (43.8)	236 (48.2)	275 (40.7)	0.013
Comorbidities, yes	555 (47.6)	238 (48.6)	317 (46.9)	0.612
Current cigarette smokers	62 (5.3)	22 (4.5)	40 (5.9)	0.562
Current alcohol drinkers	362 (31.0)	136 (27.8)	226 (33.4)	0.045
FH of thyroid cancer	159 (13.6)	65 (13.3)	94 (13.9)	0.820
Currently have a job, yes	786 (67.4)	328 (66.9)	458 (67.8)	0.819
Primary tumor size	0.8 ± 0.5^3^	0.8 ± 0.4^3^	0.8 ± 0.5^3^	0.433
≤1 cm	870 (74.6)	360 (73.5)	510 (75.4)	0.486
> 1 cm	296 (25.4)	130 (26.5)	166 (24.6)	
Extra-thyroidal extension				0.667
Microscopic	496 (42.5)	201 (41.0)	295 (43.6)	
Gross	5 (0.4)	2 (0.4)	3 (0.4)	
Cervical LN metastasis				0.741
N0/Nx	138 (11.8)	54 (11.0)	84 (12.4)	
N1a	703 (60.3)	300 (61.2)	403 (59.6)	
N1b	325 (27.9)	136 (27.8)	189 (28.0)	
TNM stage (8^th^ )				0.730
Stage I	1090 (93.5)	460 (93.9)	630 (93.2)	
Stage II	76 (6.5)	30 (6.1)	46 (6.8)	
LT4				< 0.001
LT4 users	491 (42.1)	245 (50.0)	246 (36.4)	
LT4 non-users	675 (57.9)	245 (50.0)	430 (63.6)	
TSH group & LT4				
Low-TSH group				< 0.001
LT4 users	313 (53.6)	149 (62.3)	164 (47.5)	
LT4 non-users	271 (46.4)	90 (37.7)	181 (52.5)	
High-TSH group				< 0.001
LT4 users	178 (30.6)	96 (38.3)	82 (24.8)	
LT4 non-users	404 (69.4)	155 (61.8)	249 (75.2)	
LT4 dose at 3-month FU (µg/day)^3^				
Total	45.1 ± 17.5	46.8 ± 17.6	43.5 ± 17.4	0.022
Low-TSH group	44.1 ± 17.5	46.1 ± 18.3	42.4 ± 16.7	0.065
High-TSH group	46.9 ± 17.4	48.1 ± 16.4	45.6 ± 18.5	0.349

*Abbreviations*: *PTC* Papillary thyroid carcinoma, *CAM* Complementary and Alternative Medicine, *MASTER* A Multicenter, Randomized, Controlled Trial for Assessing the Usefulness of Suppressing Thyroid Stimulating Hormone Target Levels after Thyroid Lobectomy in Low to Intermediate Risk Thyroid Cancer Patients, *LN* Lymph node, *TNM* Tumor-node-metastasis, *LT4* Levothyroxine, *BMI* Body mass index, *TSH* Thyrotropin, *FH* Family history, *SD* Standard deviation

^1^CAM users are those who have used CAM at least once during the periods from the time of thyroid cancer diagnosis to the 12-month follow-up time in the MASTER trial

^2^*P*-value for the difference between non-CAM users and CAM users by Pearson’s chi-square test or Student’s T-test

^3^Mean ± SD

Obesity (BMI ≥ 25 kg/m^^2^) prevalence was lower in CAM users (40.7%) than in non-CAM users (48.2%) (*P =* 0.013). Current alcohol drinkers were more prevalent among CAM users (33.4%) than non-users (27.8%) (*P =* 0.045). LT4 non-users (63.6%) had used CAM more than LT4 users (36.4%) (*P <* 0.001), and this observation was the same within each randomly assigned group (High-TSH group, and Low-TSH group, *P* < 0.001). In contrast to the LT4 usage proportion, LT4 dose was observed to be lower in CAM users (*P* = 0.022), and this was similarly observed when stratified by randomization group.

### CAM usage and costs

We analyzed according to the FU time and as the results, most of CAM use (%) steeply decreased from baseline to 12-month and there was only 1 case of new start from 12 months (1 out of 1,166 people). Therefore, it is expected that it is rare to start CAM after 1 year, which is the period of stabilization after thyroid cancer lobectomy.

The total cost and experience of CAM use by modality and the number of CAM modalities used are summarized in Supplementary Table 2. Among CAM users, dietary nutritional products were the most frequently used modality (66.1%), while pharmacotherapy was the least utilized (8.9%). Costs varied significantly by CAM modality, with physical therapy and exercise showing the highest median costs (288 USD). Additionally, the number of CAM modalities used was associated with increasing costs, with a median total cost of 596 USD for those using three or more modalities (Supplementary Table 2).

**Fig. 1:**
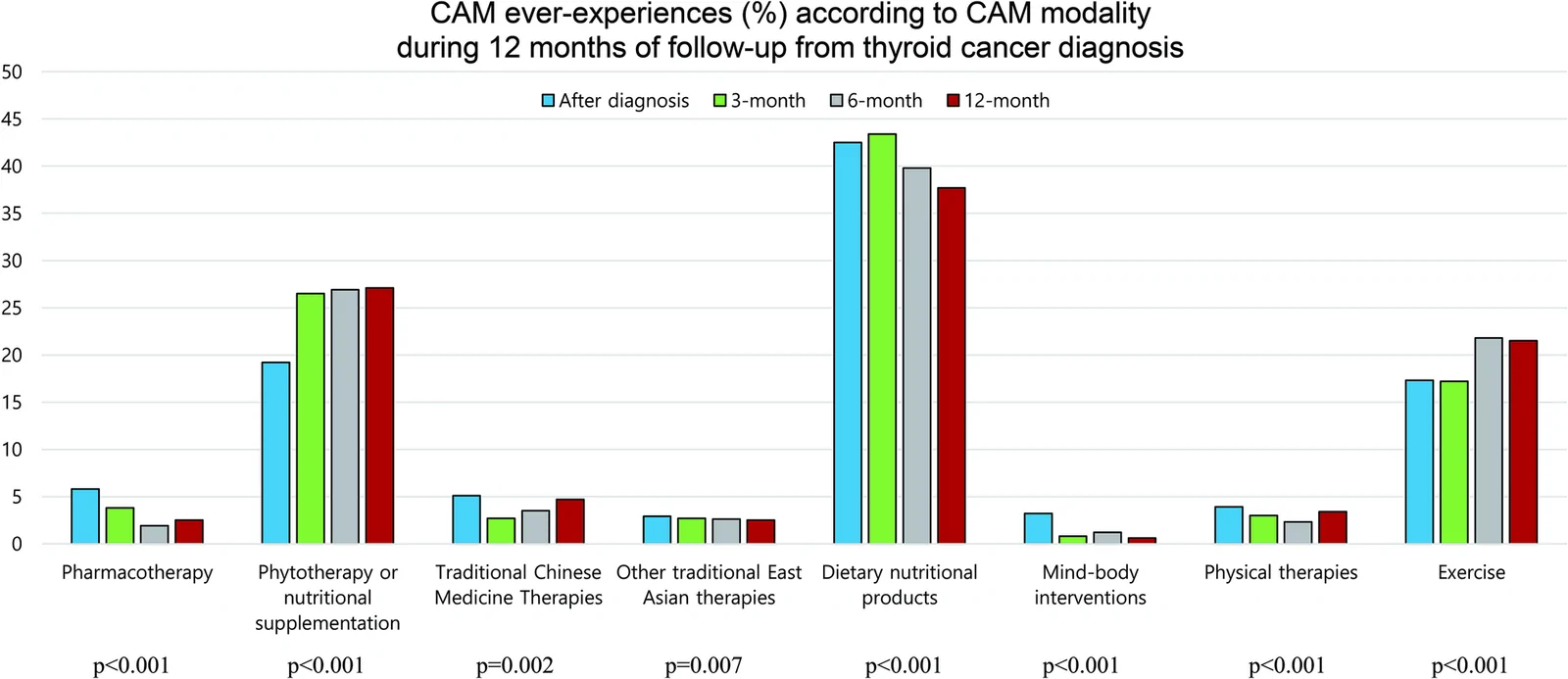
Usage rates of CAM modalities over time. Dietary nutritional products were the most used, decreasing from 42.5% at baseline to 37.7% at 12 months. Phytotherapy increased from 19.2% to 27.1%. Traditional Chinese medicine therapy use ranged from 2.5% to 5.1%. Mind-body intervention use decreased from 3.2% to 0.6%

“Dietary nutritional products” was the most frequently used CAM modality, with usage rates of 42.5% at baseline, decreasing slightly to 37.7% by 12 months. The usage rates of phytotherapy or nutritional supplementation usage increased from 19.2% at baseline to 27.1% at 12 months, whereas those for traditional Chinese medicine or traditional East Asian therapies ranged from 2.5% to 5.1%. Use of mind-body interventions and physical therapies was less frequent, dropping from 3.2% at baseline to 0.6% at 12 months. (Fig. 1 and Supplementary Table 3)

Costs associated with CAM modalities varied over time. The overall average cost remained relatively stable throughout the follow-up time points (baseline, 3 months, 6 months, and 12 months post-lobectomy), with no significant increase or decrease observed (all 144 USD, *P* = 0.39). However, specific CAM modalities, such as Pharmacotherapy, showed a noticeable cost increase at 12 months. For pharmacotherapy, median costs at baseline were 54 USD (KRW 75,000), which increased significantly to 1,151 USD (KRW 1,600,000) at 12 months. Costs for phytotherapy or nutritional supplementation remained consistent, with a median of 54 USD (KRW 75,000) at baseline and 12 months, peaking slightly at 99 USD (KRW 138,000) at 6 months. Additionally, the costs for traditional Korean medicine therapy were the highest at baseline and 3 months (288 USD or KRW 400,000) and reduced to 81 USD (KRW 113,000) by 12 months. A stable median cost of 144 USD (KRW 200,000) was maintained throughout the study period for dietary nutritional products. Mind-body interventions, initially not recorded, showed varying costs, reaching 144 USD (KRW 200,000) by 12 months. Physical therapy costs remained relatively high, starting at 288 USD (KRW 400,000) and showing fluctuations, ending again at 288 USD by 12 months. Exercise costs increased from 144 USD (KRW 200,000) at baseline to 288 USD (KRW 400,000) at later points. Detailed information is provided in Table 2.

**Table 2:** Cost ($) by the number and the type of CAM modalities at each survey point in the MASTER trial

	After diagnosis	3-month	6-month	12-month	Diff ^1^ 0–12 months ($)	
	Median (Range)	Median (Range)	Median (Range)	Median (Range)		*p*-value^3^
Type of CAM modalities^2^						
Overall average	144 (18 − 1,728)	144 (18 − 1,584)	144 (18 − 2,160)	144 (1–3,420)	0	0.04
Pharmacotherapy	54 (18–540)	54 (18–540)	54 (18–288)	1,151 (144-2,158)	1,099	0.24
Phytotherapy or nutritional supplementation	54 (18–180)	54 (18–180)	54 (18 − 1,439)	54 (18–144)	0	0.67
Traditional Korean Medicine therapies	288 (54–288)	288 (54–288)	288 (144–468)	81 (18 − 1,439)	-207	0.93
Other traditional oriental therapies	144 (144–144)	144 (144–144)	288 (108–540)	144 (18–540)	0	0.83
Dietary nutritional products	144 (18–540)	144 (18–540)	144 (18–540)	144 (18–540)	0	0.30
Mind-body interventions	NA	NA	18 (18–18)	144 (144–144)	144	NA
Physical therapies	288 (288–432)	288 (288–432)	144 (54–288)	288 (1–1,439)	5	0.25
Exercise	144 (18 − 1,439)	144 (18 − 1,439)	144 (18 − 1,439)	288 (18 − 1,439)	144	0.17
Number of CAM use						
1	144 (18 − 1,439)	144 (18 − 1,439)	144 (18 − 2,158)	144 (18 − 1,439)	0	0.14
2	234 (72 − 1,727)	288 (36 − 1,583)	288 (72 − 1,583)	297 (1–2,176)	63	0.68
3+	441 (360–594)	468 (54–630)	324 (126–342)	729 (342-3,417)	288	0.14

*Abbreviations*: *CAM* Complementary and Alternative Medicine, *MASTER* A Multicenter, Randomized, Controlled Trial for Assessing the Usefulness of Suppressing Thyroid Stimulating Hormone Target Levels after Thyroid Lobectomy in Low to Intermediate Risk Thyroid Cancer Patients

^1^CAM ever-users are those who have used CAM at least once during the periods from the time of thyroid cancer diagnosis to the 12-month follow-up time in the MASTER trial

^2^Multiple choice is allowed

^3^*p*-value for the trend of cost of CAM modalities between each survey point by using generalized linear regression

### Usage of multiple CAM modalities

**Fig. 2:**
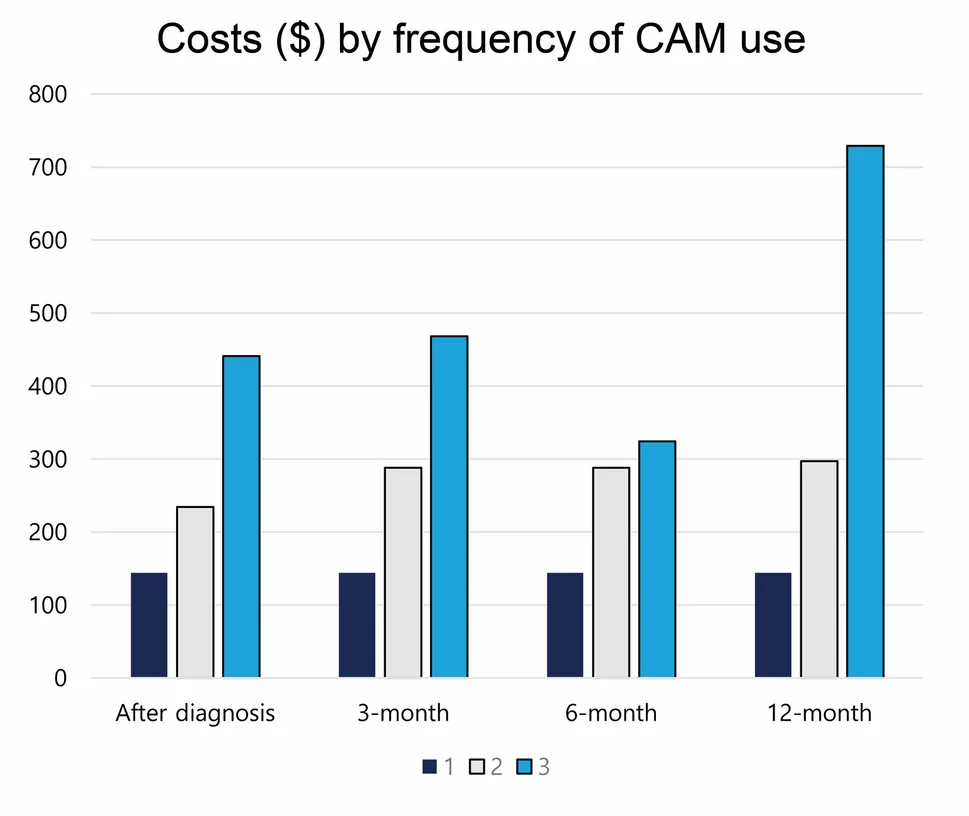
CAM experiences (%) and costs (US dollar [$]) by frequency of CAM use at each survey time point. At baseline, 70.2% of participants used one CAM modality, and this percentage remained relatively stable over time. The proportion of participants using two CAM modalities increased slightly over time (18.1% at baseline to 25.9% at 12 months). Conversely, the proportion of participants using three or more CAM modalities decreased (11.7% at baseline to 2.6% at 12 months). Participants using two CAM modalities showed a slight increase over time (18.1% at baseline to 25.9% at 12 months). Conversely, the use of three or more CAM modalities decreased over time (11.7% at baseline to 2.6% at 12 months). For those using three or more CAM modalities, costs were highest ($441 (KRW 613,000) at baseline to $729 (KRW 1,013,000) at 12 months)

Figure 2 illustrates the CAM costs (USD [$]) by the frequency of CAM use. At baseline, 70.2% of participants used one CAM modality, and this percentage remained stable over time (73.7% at 3 months, 72.5% at 6 months, and 71.4% at 12 months), with a consistent median cost of 144 USD (KRW 200,000). The proportion of participants using two CAM modalities increased from 18.1% at baseline to 25.9% at 12 months, with median costs rising from 234 USD (KRW 325,000) to $297 (KRW 413,000). While the proportion of patients using three or more CAM modalities decreased from 11.7% at baseline to 2.6% at 12 months, the costs for this group were the highest, increasing from 441 USD (KRW 613,000) to 729 USD (KRW 1,013,000). Supplementary Fig. 2 illustrates detailed the CAM experiences (%) by frequency of CAM use.

### Impact of levothyroxine, and TSH levels on CAM use

Post-hoc analyses were performed to investigate the frequency of CAM use according to TSH levels and LT4 use (Table 3). Although patients in the low TSH group showed a trend toward higher CAM use at baseline (OR: 1.28, 95% CI: 0.99–1.66, *P* = 0.06) and 3 months (OR: 1.29, 95% CI: 1.001–1.67, *P* = 0.05), this association was not statistically significant. This effect diminished over time, becoming non-significant at the 12-month mark (OR: 1.13, 95% CI: 0.85–1.50, *P* = 0.40). Low TSH levels did not significantly affect the use of three or more CAMs compared with the use of one or two CAMs at any time point (e.g., baseline: OR: 1.26, 95% CI: 0.64–2.47, *P* = 0.50).

**Table 3:** Risk factors affecting CAM use relative to non-use or 3 more CAM use relative to 1–2 CAM use among CAM users in the MASTER trial (multivariable analysis)

		Non-use	use			1–2 CAM use	3 + CAM use		
		*N* (%)	*N* (%)	OR (95% CI)^1^	*p*-value^1^	*N* (%)	*N* (%)	OR (95% CI)^2^	*p*-value^2^
Baseline	Younger age (< 60 years)	639 (82.7)	320 (81.4)	0.88 (0.64–1.22)	0.44	287 (82.7)	33 (71.7)	0.45 (0.21–0.95)	0.04
	Female	590 (76.3)	313 (79.6)	1.34 (0.98–1.84)	0.07	274 (79.0)	39 (84.8)	1.44 (0.58–3.57)	0.44
	BMI ≥ 25 kg/m^2^	345 (44.6)	166 (42.2)	0.93 (0.72–1.21)	0.60	149 (42.9)	17 (37.0)	0.74 (0.38–1.47)	0.39
	Current alcohol drinkers	225 (29.1)	137 (34.9)	1.35 (1.03–1.78)	0.03	124 (35.7)	13 (28.3)	1.05 (0.50–2.19)	0.90
	LT4 use	366 (47.4)	125 (31.8)	0.49 (0.38–0.64)	< 0.001	98 (28.2)	27 (58.7)	3.66 (1.88–7.14)	0.001
	Low TSH	383 (49.6)	201 (51.2)	1.28 (0.99–1.66)	0.06	173 (49.9)	28 (60.9)	1.26 (0.64–2.47)	0.50
3-month	Younger age (< 60 years)	629 (81.7)	330 (83.3)	1.09 (0.78–1.51)	0.62	309 (82.6)	21 (95.5)	4.58 (0.59–35.22)	0.14
	Female	588 (76.4)	315 (27.0)	1.26 (0.92–1.73)	0.15	296 (79.1)	19 (86.4)	1.64 (0.43–6.26)	0.47
	BMI ≥ 25 kg/m^2^	350 (45.5)	161 (40.7)	0.84 (0.65–1.09)	0.19	152 (40.6)	9 (40.9)	1.22 (0.48–3.09)	0.67
	Current alcohol drinkers	228 (29.6)	134 (33.8)	1.23 (0.94–1.62)	0.14	128 (34.2)	6 (27.3)	0.82 (0.30–2.25)	0.70
	LT4 use	356 (46.2)	135 (34.1)	0.57 (0.44–0.74)	< 0.001	123 (32.9)	12 (54.6)	1.96 (0.78–4.91)	0.15
	Low TSH	378 (49.1)	206 (52.0)	1.29 (1.00-1.67)	0.05	191 (51.1)	15 (68.2)	1.69 (0.65–4.42)	0.29
6-month	Younger age (< 60 years)	684 (81.3)	275 (84.6)	1.23 (0.86–1.75)	0.26	263 (84.3)	11 (91.7)	1.80 (0.22–14.75)	0.58
	Female	642 (76.3)	261 (80.3)	1.32 (0.94–1.85)	0.11	253 (81.1)	8 (66.7)	0.70 (0.18–2.76)	0.61
	BMI ≥ 25 kg/m^2^	381 (45.3)	130 (40.0)	0.84 (0.64–1.10)	0.20	121 (38.8)	8 (66.7)	2.89 (0.81–10.34)	0.10
	Current alcohol drinkers	252 (30.0)	110 (33.9)	1.21 (0.91–1.61)	0.20	104 (33.3)	5 (41.7)	1.19 (0.34–4.22)	0.79
	LT4 use	383 (45.5)	108 (33.2)	0.54 (0.41–0.72)	< 0.001	103 (33.0)	4 (33.3)	0.98 (0.26–3.64)	0.98
	Low TSH	409 (48.6)	175 (53.9)	1.42 (1.09–1.86)	0.01	167 (53.5)	8 (66.7)	1.78 (0.50–6.37)	0.38
12-month	Younger age (< 60 years)	725 (80.7)	234 (87.3)	1.72 (1.15–2.57)	0.01	226 (87.3)	6 (85.7)	0.60 (0.07–5.55)	0.66
	Female	681 (75.8)	222 (82.8)	1.47 (1.01–2.14)	0.04	215 (83.0)	5 (71.4)	0.77 (0.10–5.82)	0.80
	BMI ≥ 25 kg/m^2^	410 (45.7)	101 (37.7)	0.78 (0.58–1.04)	0.09	98 (37.8)	3 (42.9)	0.96 (0.17–5.39)	0.96
	Current alcohol drinkers	283 (31.5)	79 (29.5)	0.89 (0.65–1.22)	0.48	74 (28.6)	4 (57.1)	3.95 (0.71–22.09)	0.12
	LT4 use	400 (44.5)	91 (34.0)	0.60 (0.44–0.80)	0.001	86 (33.2)	3 (42.9)	2.79 (0.44–17.50)	0.27
	Low TSH	448 (49.9)	136 (50.8)	1.13 (0.85–1.50)	0.40	131 (50.6)	3 (42.9)	0.52 (0.09–3.04)	0.47

*Abbreviations*: *CAM *Complementary and Alternative Medicine, *MASTER* A Multicenter, Randomized, Controlled Trial for Assessing the Usefulness of Suppressing Thyroid Stimulating Hormone Target Levels after Thyroid Lobectomy in Low to Intermediate Risk Thyroid Cancer Patients, *OR* Odds ratio, *CI* Confidence interval, *LR* Logistic regression, *TSH* Thyroid-stimulating hormone, *LT4* Levothyroxine, *BMI* Body mass index

^1^Adjusted for age (< 60 vs. ≥60 years old(reference)), sex (female vs. male(reference)), BMI (≥ 25 kg/m^2^ vs. <25 kg/m^2^ (reference)), alcohol drinking (current drinking vs. never drinking (reference)), TSH level (low vs. high(reference)), and levothyroxine use (use vs. non-use(reference)) on use of CAMs rather than non-use of CAMs

^2^Adjusted for age (< 60 vs. ≥60 years old(reference)), sex (female vs. male(reference)), BMI (≥ 25 kg/m^2^ vs. <25 kg/m^2^ (reference)), alcohol drinking (current drinking vs. never drinking (reference)), TSH level (low vs. high(reference)), and levothyroxine use (use vs. non-use(reference)) on use of 3 or more CAMs rather than use of 1 or 2 CAMs

**Fig. 3:**
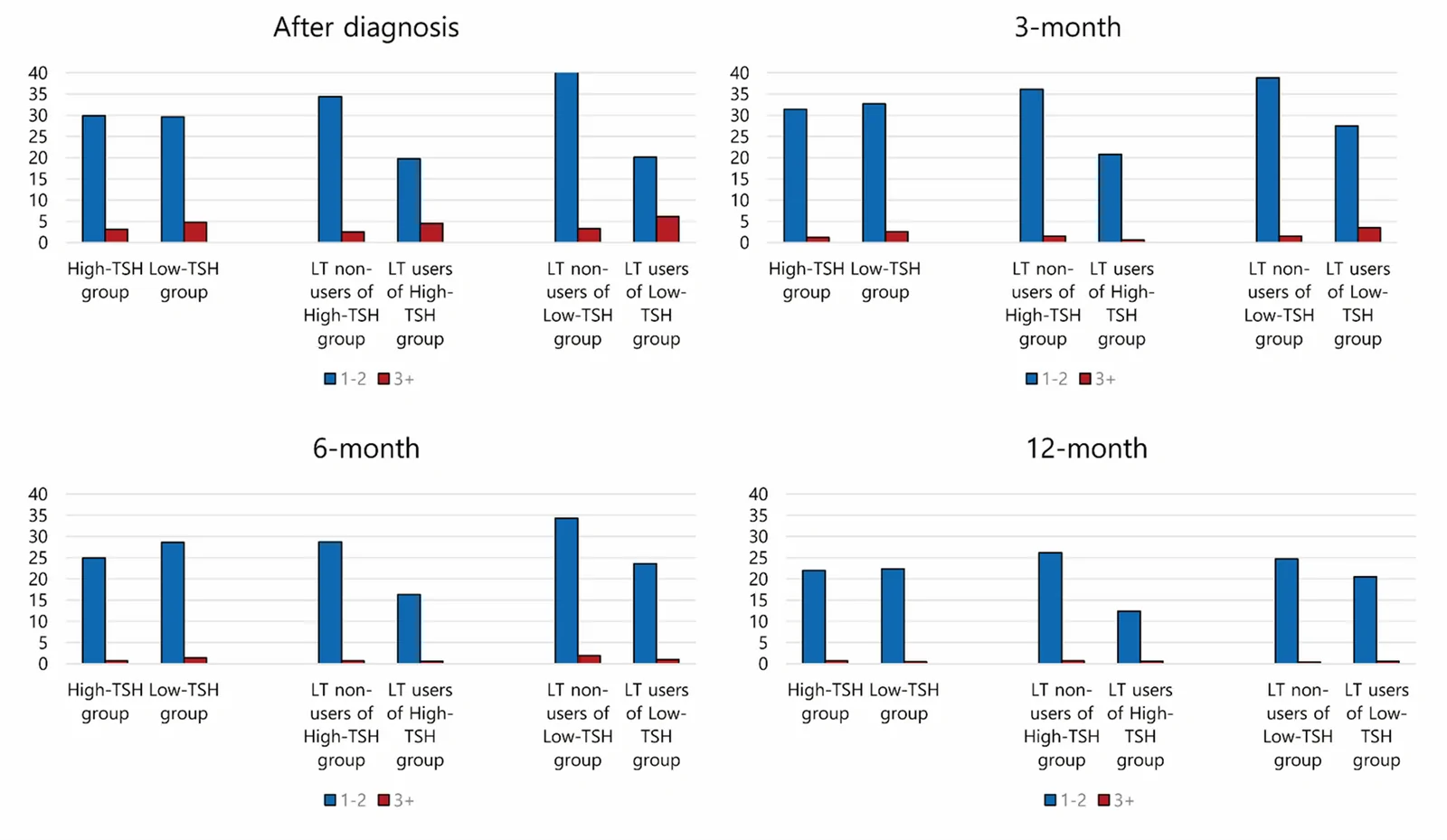
CAM experiences (%) by the number of CAM modalities among CAM users between high-TSH and low-TSH group at various time points. This figure highlights the differences in CAM usage patterns between high-TSH and low-TSH groups and shows the impact of LT4 use on the frequency of CAM usage over time. The trends suggest a higher tendency towards multiple CAM use among LT4 users in the low-TSH group, particularly at baseline and at 3-month follow-up

In contrast, LT4 use consistently reduced the likelihood of CAM use across all time points (baseline: OR: 0.49, 95% CI: 0.38–0.64, *P* < 0.001; 12-month: OR: 0.60, 95% CI: 0.44–0.80, *P* = 0.006). LT4 users were more likely to use three or more CAMs than one or two CAMs, especially at the baseline (OR: 3.66, 95% CI: 1.88–7.14, *P <* 0.001). In the high TSH group, LT4 use reduced the likelihood of CAM use (baseline: OR: 0.56, 95% CI: 0.37–0.83, *P* = 0.004; 6-month: OR: 0.50, 95% CI: 0.32–0.78, *P* = 0.002). LT4 use in the high TSH group did not affect the likelihood of using three or more CAMs compared with using one or two CAMs at most time points. In the low TSH group, LT4 use also reduced the likelihood of CAM use (baseline: OR: 0.46, 95% CI: 0.32–0.65, *P* < 0.001; 6-month: OR: 0.58, 95% CI: 0.41–0.84, *P* = 0.003). LT4 users in the low TSH group were more likely to use three or more CAMs compared with one or two CAMs at the 3-month mark (OR: 3.58, 95% CI: 1.06–12.09, *P* = 0.04); however, this effect was not consistent across time points. Detailed information on the frequency of CAM use according to TSH group and LT4 usage is provided in Supplementary Tables 4, 5 and Fig. 3.

Among CAM users, the type of CAM modalities used varied significantly between LT4 users and LT4 non-users (Table 4). Phytotherapy or Nutritional Supplementation was the most used modality among LT4 users (72.8%) compared to LT4 non-users (48.6%), showing a statistically significant difference (*P* < 0.01). Traditional Korean Medicine Therapies and Other Traditional Oriental Therapies were also more frequently used among LT4 users (38.2% for both) than LT4 non-users (30.0% and 27.4%, respectively), indicating a preference for traditional Eastern practices in this group (*P* = 0.03 and *P* < 0.01, respectively).Mind-body Interventions (35.8% vs. 25.6%, *P* = 0.01) and Physical Therapies (35.0% vs. 27.2%, *P* = 0.03) were significantly more prevalent among LT4 users. In contrast, Dietary Nutritional Products were less frequently used by LT4 users (64.6%) compared to LT4 non-users (74.4%), though the difference remained statistically significant (*P* = 0.01). Pharmacotherapy and Exercise showed no significant differences between the two groups (*P* = 0.13 and *P* = 0.22, respectively).

**Table 4:** Types of CAM modalities used with levothyroxine among CAM users

	CAM ever-users^1^ (*n* = 676)	LT4 non-users (*n* = 430)		LT4 users (*n* = 246)	
	*N* (%)	*N* (%)	*N* (%)		*p*-value^3^
Type of CAM modalities^2^					
Pharmacotherapy	60 (8.9)	126 (29.3)	86 (35.0)		0.13
Phytotherapy or nutritional supplementation	294 (43.5)	209 (48.6)	179 (72.8)		< 0.01
Traditional Korean Medicine therapies	67 (9.9)	129 (30.0)	94 (38.2)		0.03
Other traditional oriental therapies	51 (7.5)	118 (27.4)	94 (38.2)		< 0.01
Dietary nutritional products	447 (66.1)	320 (74.4)	159 (64.6)		0.01
Mind-body interventions	26 (3.8)	110 (25.6)	88 (35.8)		0.01
Physical therapies	51 (7.5)	117 (27.2)	87 (35.0)		0.03
Exercise	240 (35.5)	201 (46.7)	127 (51.6)		0.22
Number of CAM use					
1	337 (49.9)	227 (52.8)	110 (44.7)		< 0.01
2	202 (29.9)	138 (32.1)	64 (26.0)		
3+	137 (20.2)	65 (15.1)	72 (29.3)		
1	337 (49.9)	227 (52.8)	110 (44.7)		< 0.01
2	202 (29.9)	138 (32.1)	64 (26.0)		
3	85 (12.6)	39 (9.1)	46 (18.7)		
4	32 (4.7)	19 (4.4)	13 (5.3)		
5	12 (1.8)	5 (1.2)	7 (2.9)		
6+	8 (1.2)	2 (0.5)	6 (2.4)		

*Abbreviations*: *CAM* Complementary and Alternative Medicine, *MASTER* A Multicenter, Randomized, Controlled Trial for Assessing the Usefulness of Suppressing Thyroid Stimulating Hormone Target Levels after Thyroid Lobectomy in Low to Intermediate Risk Thyroid Cancer Patients

^1^CAM ever-users are those who have used CAM at least once during the periods from the time of thyroid cancer diagnosis to the 12-month follow-up time in the MASTER trial

^2^Multiple choice is allowed

^3^The *p*-value for the difference between LT4 non-users and LT4 users by Pearson’s chi-square test

### Other risk factors affecting CAM use

Younger patients (age ≤ 60 years) initially did not show a significant association with CAM use but showed a trend toward higher CAM use at 12 months (OR 1.72, 95% CI 1.15–2.57, *P* = 0.01). Although younger age was associated with a lower likelihood of using three or more CAMs at baseline (OR 0.45, 95% CI 0.21–0.95, *P* = 0.04), this was not significant in later follow-ups. Females were consistently more likely to use CAM, with a marginal association at baseline (OR 1.34, 95% CI 0.98–1.84, *P* = 0.07) that became significant at 12 months (OR 1.47, 95% CI 1.01–2.14, *P* = 0.04). Female sex was also associated with a higher rate of multiple CAMs use, although no statistically significant difference was observed at any time point. Higher BMI was not significantly associated with CAM use at any time point and did not influence the likelihood of using three or more CAMs compared with using one to two CAMs. Current alcohol drinkers were more likely to use CAM at baseline (OR 1.35, 95% CI 1.03–1.78, *P* = 0.03); however, this association was not significant in the following time points. Alcohol consumption did not significantly affect the likelihood of using multiple CAMs at any time point. (Table 3 and Supplementary Table 6)

## Discussion

Our study revealed that 58% of participants used CAM, with nearly half utilizing multiple types. There were no significant differences in age, sex distribution, primary tumor size, extrathyroidal extension, cervical lymph node metastasis status, or TNM staging between CAM and non-CAM users. Interestingly, CAM users had a significantly lower prevalence of obesity than non-users. Dietary nutritional products were the most popular, and exercise had the highest median cost among the various CAM modalities. The economic burden associated with CAM was substantial, with costs varying widely by modality. The median cost for CAM ranged from 54 USD (KRW 75,000) to over 1,151 USD (KRW 1,600,000) depending on the therapy, highlighting the financial implications of CAM use. Additionally, logistic regression analysis suggested levothyroxine users and patients with obesity were less likely to use CAM. Females tended to use multiple CAM modalities. Females tended to use multiple CAM modalities.

The results of this study elucidate the prevalent use of CAM among patients with PTC, highlighting a significant proportion (58%) engaging in CAM, with almost half exploring more than one type. Despite the difference in study methods, studies reported that the proportion of CAM users among patients with cancer ranged from 34% to 79% [7, 8, 10, 16, 17]. In a study conducted on patients with thyroid cancer in the US, the top five CAM modalities were massage therapy, chiroprax, special diets, herbal tea, and yoga [10]. In contrast, dietary nutritional products, nutritional supplementation, exercise, or traditional Chinese medicine therapy were commonly used CAM modalities in our study. These modalities appear at similar rates in studies targeting Korean patients with cancer [17]. Therefore, these findings suggest that CAM use is influenced more by cultural and social factors than by the specific disease itself. Previous studies have shown that CAM preferences differ by region, reflecting differences in healthcare practices and patient attitudes [26, 27]. To gain deeper insight into these influences, future research should include qualitative approaches such as patient interviews and focus groups.

The cost associated with CAM use presents a substantial financial burden, with median expenditures varying across different modalities. Our findings suggest that cost variations among CAM modalities are likely influenced by both changes in usage frequency and the nature of specific therapies. While overall CAM use increased over time (*p* < 0.001), some modalities, such as pharmacotherapy, showed a significant rise in costs despite stable usage. This suggests that patient demand and external factors, such as pricing changes and treatment complexity, may also contribute to cost differences. Future research should further investigate these factors to better understand the economic impact of CAM use in thyroid cancer patients. For instance, the median cost for pharmacotherapy ranged from 54 USD (KRW 75,000) at baseline to 1,151 USD (KRW 1,600,000) at 12 months, whereas that of dietary nutritional products was consistent at 144 USD (KRW 200,000) across all time points. In a 2019 study involving 1297 Swedish patients with cancer, approximately 67% reported spending < 50 USD dollars [7]. In a 2016 study on cancer survivors in the US, the annual average spent was 445 USD, which is approximately 525 USD in 2024 [16]. In contrast, according to a study on Korean patients with cancer in 2004, the average monthly cost of CAM was 1,380,000 won (adjusted to approximately 2,207,948 KRW or approximately 1,698 USD). Additionally, 45.2% of CAM users paid less than 799,977 KRW (or approximately 615 USD) per month, whereas 19.9% paid more than 1,599,953 KRW (or about 1,231 USD) [17]. The costs reported from various international studies on CAM usage have been standardized to USD and adjusted for inflation to 2024 values. Although direct comparison is difficult, considering our study, it seems that Korean patients with cancer tend to pay more for CAM. This reveals the financial impact of CAM use, with considerable expenditure on some therapies. The financial burden of CAM use varies significantly across different modalities. Some therapies, such as traditional herbal medicine and specialized nutritional supplements, impose substantial costs on patients. The high out-of-pocket expenditures for CAM, which are not covered by the national health insurance system in Korea, may lead to financial strain, particularly for patients already managing the economic impact of their cancer treatment [17].This cost burden can influence patients’ decisions regarding CAM use and even impact their adherence to conventional treatments [16]. Given the increasing reliance on CAM in thyroid cancer patients, there is a need to explore its cost-effectiveness and whether policy-level discussions should consider reimbursement models for evidence-based CAM therapies [17]. Future research should further investigate patient decision-making processes related to CAM expenditure and its effect on clinical outcomes.

Previous studies, regardless of whether they were Western or Korean, have found that individuals with higher levels of education, younger age, and female sex are more likely to use CAM [7, 8, 10, 17]. In our study, while sex and age did not seem to be related to CAM use at most survey points, younger and female patients were more likely to use CAM modalities, particularly at the 12-month mark. This may be due to the characteristics of the study population, as this was a prospective study on thyroid cancer. As the postoperative period lengthens, younger and female patients appear to be more inclined to seek treatments beyond conventional therapies.

In our study, patients on levothyroxine were less likely to use CAM, but once they started, they tended to use multiple CAM modalities. While we did not collect direct data on patient concerns about interactions between CAM and levothyroxine, the literature suggests that patients on conventional therapies often have such concerns [7, 28]. CAM users are highly engaged in their health management and concerned about side effects from conventional treatments [28]. Our observation that LT4 users were generally less likely to use CAM but tended to use multiple CAM modalities when they opted to use CAMs is consistent with previous results. Patients on LT4 likely feel their condition is adequately managed, making them less inclined to seek additional treatments [28]. Conversely, patients not on LT4 might perceive their thyroid status as inadequate, leading to higher TSH levels and increased CAM use. Our analysis showed that the association between low TSH levels and CAM use was not consistently significant across time points, as indicated by confidence intervals crossing 1. While there was an initial trend toward increased CAM use in the low TSH group, this effect diminished over time. Since TSH levels were randomly assigned in this study, patient-driven decisions did not influence these groupings. The observed differences in CAM use likely reflect patient perceptions of their treatment adequacy rather than a direct effect of TSH levels themselves. LT4 users who frequently adjust their dosage might perceive instability in their treatment, prompting more CAM use. While we did not directly determine the reasons, previous findings suggest that patients with chronic conditions are more likely to use multiple CAM modalities, which might explain our results [28, 29]. Our findings indicate that CAM users who take levothyroxine are more likely to engage in multiple CAM modalities, particularly preference for traditional Eastern practice, Mind-body Interventions, and Physical Therapies. This trend may reflect a complementary approach to hormone replacement therapy, where patients seek additional support for fatigue, immunity, and overall well-being [30, 31]. In East Asian cultures, traditional therapies are perceived to enhance energy and balance, which may explain the preference for oriental therapies among LT4 users [32, 33]. Further studies are needed to explore the psychological and cultural drivers behind this pattern. Nevertheless, our study does not conclusively prove the hypothesis, highlighting the need for further research to confirm these findings. Future research should further explore the psychological and behavioral factors influencing CAM adoption in this population.

Our study had some limitations. First, the study’s reliance on self-reported data might introduce bias as patients could overestimate or underestimate their use of CAM therapies. However, it is important to note that the accuracy of the collected data was expected to be high, as most surveys were conducted with medical professionals, specifically research nurses. These healthcare professionals actively assisted the participants in understanding the questions and provided clarifications as needed, thereby reducing the likelihood of misinterpretation and ensuring more reliable and accurate responses. In addition, the questionnaire was not independently psychometrically validated, and no pilot testing was conducted. Therefore, the findings should be interpreted as simple descriptive estimates of CAM use within this study population, as the questionnaire was not validated for broader use. Regardless, only 1,166 of 2,407 patients completed the CAM questionnaire, raising the possibility of selection bias. A comparative analysis between responders and non-responders (Supplementary Table 1) showed that most demographic and clinical characteristics, including LT4 usage, were generally comparable between the two groups. However, modest but statistically significant differences in sex distribution and BMI suggest a potential selection bias that may limit the generalizability of our findings on CAM use. Additionally, information bias such as recall bias or memory decay could not be excluded because this study investigated CAM use over the past 3 months for thyroid cancer management. Furthermore, as the survey was conducted in a clinical setting, patients might have been reluctant to disclose their use of CAM due to concerns about practitioners’ judgment, which could have led to underreporting and underestimation of the true prevalence of CAM use [9]. Second, the random allocation was performed before lobectomy, so the CAM survey at the baseline could investigate patients’ experience of using CAM from the time of thyroid cancer diagnosis until lobectomy. Thus, we believe that CAM use will not have influenced the decision to lobectomy, nor will it have influenced the randomization (high-TSH or low-TSH group). Additionally, because LT4 use is determined by the effect of TSH suppression, CAM use will not affect the decision to use LT4. However, LT4 use at baseline may be determined before CAM use, so the reverse causation problem cannot be avoided at the baseline. Specifically, the nature of the cross-sectional data limited our ability to draw causal inferences for those patients who were already using CAM at the baseline. However, for patients who began using CAM after the baseline survey, the longitudinal data from subsequent time points (3, 6, 12 months) provide a clearer picture of the relationship between CAM use and health outcomes following the thyroid cancer diagnosis. Third, the focus of this study on a specific patient population may limit the generalizability of the findings to other cancer types and broader populations. In this study, the CAM questionnaire was designed to ensure that patients responded only about their use of CAM for the purpose of thyroid cancer management. We did not investigate other objectives for CAM use in the study. Therefore, this will have relatively little or low impact in limiting the generalizability to the PTC population. Fourth, owing to the small number of users for some CAMs, detailed analysis of clinical characteristics for each type of CAM was challenging, indicating a need for further research in this area. In addition, at present, our study does not include an evaluation of CAM’s impact on thyroid cancer outcomes. Although this study does not evaluate the effects of CAM on thyroid cancer outcomes, recurrence-free survival is the primary outcome of the MASTER study. Future analyses may assess whether CAM usage has any impact on disease progression or patient quality of life. Further research is warranted to determine if CAM plays a role in clinical outcomes for thyroid cancer patients. Finally, the lack of blinding and varying doses of LT4 in our study could introduce biases, and future studies should consider these aspects to mitigate potential confounding factors.

In conclusion, our study on the use of CAM by patients with thyroid cancer highlighted that over half (58%) used CAM, with many choosing multiple forms. This underscores the importance of integrating CAM into the healthcare system. Economic analysis revealed significant spending on CAM, especially on dietary supplements and exercise, indicating a considerable financial burden. The data suggest a need for healthcare professionals to engage more in CAM discussions to ensure patient safety and integrate CAM effectively into care plans. Further research is required to evaluate CAM’s efficacy and cost-effectiveness, aiming to guide both patients and practitioners in making informed decisions regarding its use in cancer care.

## Supplementary Information

Supplementary Material 1.

## Data Availability

The datasets generated during and/or analyzed in the current study are available from the corresponding author upon reasonable request.

## Declarations

A portion of this study was presented in abstract form at the AOCE-SICEM 2023 (Asia Oceanian Congress of Endocrinology and Seoul International Congress of Endocrinology and Metabolism 2023) in Seoul, Korea.
