# Supplementary material for: Patterns of complementary and alternative medicine use and factors influencing its utilization in thyroid cancer: findings from the MASTER study
Source: BMC Complement Med Ther. 2026 Jun 23;26:264. doi: 10.1186/s12906-026-05428-w (PMC13543374; doi:10.1186/s12906-026-05428-w)
Supplement: Supplementary file 1 [file 12906_2026_5428_MOESM1_ESM.docx]

**Supplementary Tables**

Supplementary Table 1. Baseline Characteristics of Patients in the MASTER Trial: Comparison Between CAM Survey Participants and Non-Participants

Supplementary Table 2. Experience of use and total costs by CAM modality of thyroid cancer patients with experiences of CAM use from thyroid cancer diagnosis to 12-month follow-up in the MASTER trial

Supplementary Table 3. CAM experiences^1^ (%) and costs ($) by the frequency of CAM use at each survey point in the MASTER trial

Supplementary Table 4. Factors affecting CAM use or multiple use of 3 or more among CAM users in the MASTER trial (baseline, 3-month and 12-month follow-up^1^)

Supplementary Table 5. Factors affecting CAM use or multiple use of 3 or more in the two randomly assigned arms (High-TSH group and Low-TSH group) of the MASTER trial (baseline, 3-month and 12-month follow-up^1^)

Supplementary Table 6. Factors^1^ affecting CAM use or multiple use of 3 or more among CAM users in the MASTER trial

**Supplementary Figure**

Supplementary Figure 1. Flow diagram for the selection of study participants.

Supplementary Figure 2. CAM experiences by frequency of CAM use

**Supplementary Materials**

Supplement material I: MASTER Trial: Detailed Study Protocol and Eligibility Criteria

Supplemental material II. Complementary and Alternative Medicine (CAM): Survey for Thyroid Cancer patients (Initial & Follow-up)

**Supplementary Table 1. Baseline Characteristics of Patients in the MASTER Trial: Comparison Between CAM Survey Participants and Non-Participants**

| **Characteristics**  **at the baseline** | **Total patients enrolled in MASTER trial^1^**  **(n=2.407)** | **CAM survey non-participants**  **(n=1,241)** | **CAM survey participants**  **(n=1,166)** | ***P*-value^2^** |
| --- | --- | --- | --- | --- |
|  | **N (%)** | **N (%)** | **N (%)** |  |
| **Random-assigned two arms** |  |  |  | 0.59 |
| **Low-TSH group** | 1,197 (49.7) | 613 (49.4) | 584 (50.1) |  |
| **High-TSH group** | 1,210 (50.3) | 628 (50.6) | 582 (49.9) |  |
| **Age (years old)** |  |  |  | 0.93 |
| **< 40** | 680 (28.3) | 352 (28.4) | 328 (28.1) |  |
| **40 – 59** | 1,306 (54.3) | 675 (54.4) | 631 (54.1) |  |
| $\boldsymbol{\geq}$**60** | 421 (17.5) | 214 (17.2) | 207 (17.8) |  |
| **Sex, male** | 604 (25.1) | 341 (27.5) | 263 (22.6) | 0.01 |
| **BMI (kg/m^2^) ≥25 kg/m^2^ (Obesity)** | 1,007 (41.8) | 496 (40.0) | 511 (43.8) | 0.04 |
| **Comorbidities, yes** | 1,109 (46.1) | 554 (44.6) | 555 (47.6) | 0.04 |
| **Current cigarette smokers** | 182 (7.6) | 120 (9.7) | 62 (5.3) | <0.01 |
| **Current alcohol drinkers** | 769 (31.9) | 407 (32.8) | 362 (31.0) | 0.33 |
| **FH of thyroid cancer** | 340 (14.1) | 181 (14.6) | 159 (13.6) | 0.54 |
| **Primary tumor size** |  |  |  | 0.07 |
| **≤1cm** | 1,835 (76.2) | 965 (77.8) | 870 (74.6) |  |
| **>1cm** | 572 (23.8) | 276 (22.2) | 296 (25.4) |  |
| **Extra-thyroidal extension** |  |  |  | <0.01 |
| **Microscopic** | 910 (37.8) | 414 (33.4) | 496 (42.5) |  |
| **Gross** | 23 (1.0) | 18 (1.5) | 5 (0.4) |  |
| **No** | 1,474 (61.2) | 809 (65.2) | 665 (57.0) |  |
| **Cervical LN metastasis** |  |  |  | 0.04 |
| **N0/Nx** | 353 (14.7) | 215 (17.3) | 138 (11.8) |  |
| **N1a** | 1,356 (56.3) | 653 (52.6) | 703 (60.3) |  |
| **N1b** | 698 (29.0) | 373 (30.1) | 325 (27.9) |  |
| **TNM stage (8^th^)** |  |  |  | 0.47 |
| **Stage I** | 2,246 (93.3) | 1,156 (93.2) | 1,090 (93.5) |  |
| **Stage II** | 161 (6.7) | 85 (6.8) | 76 (6.5) |  |
| **LT4** |  |  |  | 0.84 |
| **LT4 users** | 1,011 (42.0) | 520 (41.9) | 491 (42.1) |  |
| **LT4 non-users** | 1,396 (58.0) | 721 (58.1) | 675 (57.9) |  |

# Abbreviations: CAM, Complementary and Alternative Medicine; MASTER, A Multicenter, Randomized, Controlled Trial for Assessing the Usefulness of Suppressing Thyroid Stimulating Hormone Target Levels after Thyroid Lobectomy in Low to Intermediate Risk Thyroid Cancer Patients, LN, lymph node; TNM, tumor-node-metastasis; LT4, levothyroxine; BMI, body mass index; TSH, thyrotropin; FH, Family history, LT4, levothyroxine.

# 1. Patients enrolled in MASTER trial until September 5 th, 2023.

# 2. P-value for the difference between non-participants and participants of CAM survey by Pearson's chi-square test.

**Supplementary Table 2 Experience of use and total costs by CAM modality of thyroid cancer patients with experiences of CAM use from thyroid cancer diagnosis to 12-month follow-up in the MASTER trial**

|  | **Total cost**  **paid, $^1^** | **CAM**  **Ever-users^2^** | **Random-assigned arm** | |  |
| --- | --- | --- | --- | --- | --- |
|  |  |  | **High-TSH group** | **Low-TSH**  **group** |  |
|  | **Median (range)** | **N (%)** | **N (%)** | **N (%)** | **p-value^4^** |
| **Type of CAM modalities^3^** |  |  |  |  |  |
| Pharmacotherapy | 81 (18-2,160) | 60 (8.9) | 32 (9.7) | 28 (8.1) | 0.566 |
| Phytotherapy or nutritional supplementation | 54 (18-1,440) | 294 (43.5) | 135 (40.8) | 159 (46.1) | 0.189 |
| Traditional Korean Medicine therapies | 144 (18-2,880) | 67 (9.9) | 32 (9.7) | 35 (10.1) | 0.937 |
| Other traditional oriental therapies | 144 (18-540) | 51 (7.5) | 22 (6.6) | 29 (8.4) | 0.472 |
| Dietary nutritional products | 144 (18-828) | 447 (66.1) | 219 (66.2) | 228 (66.1) | 0.999 |
| Mind-body interventions | 36 (18-144) | 26 (3.8) | 12 (3.6) | 14 (4.1) | 0.926 |
| Physical therapies | 288 (1-1,728) | 51 (7.5) | 21 (6.3) | 30 (8.7) | 0.312 |
| Exercise | 288 (18-2,880) | 240 (35.5) | 110 (33.2) | 130 (37.7) | 0.259 |
|  |  |  |  |  |  |
| **Number of CAM use** |  |  |  |  |  |
| 1 | 108 (7-7,200) | 337 (49.9) | 176 (53.2) | 161 (46.5) | 0.083 |
| 2 | 288 (29-4,471) | 202 (29.9) | 95 (28.7) | 107 (30.9) |  |
| 3+ | 596 (85-8,136) | 137 (20.2) | 60 (18.1) | 78 (22.6) |  |
|  |  |  |  |  |  |
| 1 | 108 (7-7,200) | 337 (49.9) | 176 (53.2) | 161 (46.7) | 0.046 |
| 2 | 288 (29-4,471) | 202 (29.9) | 95 (28.7) | 107 (31.0) |  |
| 3 | 482 (85-1,602) | 85 (12.6) | 36 (10.9) | 49 (14.2) |  |
| 4 | 758 (194-8.136) | 32 (4.7) | 16 (4.8) | 16 (4.6) |  |
| 5 | 936 (936-936) | 12 (1.8) | 4 (1.2) | 8 (2.3) |  |
| 6+ | 9,738 (882-28,800) | 8 (1.2) | 4 (1.2) | 4 (1.2) |  |

Abbreviations: CAM, Complementary and Alternative Medicine; MASTER, A Multicenter, Randomized, Controlled Trial for Assessing the Usefulness of Suppressing Thyroid Stimulating Hormone Target Levels after Thyroid Lobectomy in Low to Intermediate Risk Thyroid Cancer Patients

1. One dollar is calculated as 1,388.889 Korean won.

# 2. CAM ever-users are those who have used CAM at least once during the periods from the time of thyroid cancer diagnosis to the 12-month follow-up time in the MASTER trial.

3. Multiple choice is allowed

4. p-value for the difference between high TSH group and low TSH group by Pearson's chi-square test

**Supplementary Table 3. CAM experiences^1^ (%) and costs ($) by the frequency of CAM use at each survey point in the MASTER trial**

|  | **Survey points** | | | | | | | | |
| --- | --- | --- | --- | --- | --- | --- | --- | --- | --- |
| **CAM modalities^2^** | **After diagnosis** | | **3-month** | | **6-month** | | **12-month** | | |
|  | **N (%)** | **Median**  **(Range), $** | **N (%)** | **Median**  **(Range), $** | **N (%)** | **Median (Range), $** | | **N (%)** | **Median**  **(Range), $** |
| Pharmacotherapy | 34 (5.8) | 54 (18-540) | 20 (3.8) | 54 (18-288) | 8 (1.9) | 14 (7-72) | 9 (2.5) | | 1,151 (144-2,158) |
| Phytotherapy or nutritional supplementation | 113 (19.2) | 54 (18-180) | 140 (26.5) | 54 (18-1,439) | 115 (26.9) | 99 (18-144) | 97 (27.1) | | 54 (18-144) |
| Traditional Chinese Medicine Therapies | 30 (5.1) | 288 (54-288) | 14 (2.7) | 288 (144-468) | 15 (3.5) | 99 (18-1,439) | 17 (4.7) | | 81 (18-1,439) |
| Other traditional East Asian therapies | 17 (2.9) | 144 (144-144) | 14 (2.7) | 288 (108-540) | 11 (2.6) | 198 (108-360) | 9 (2.5) | | 144 (18-540) |
| Dietary nutritional products | 250 (42.5) | 144 (18-540) | 229 (43.4) | 144 (18-540) | 170 (39.8) | 144 (18-540) | 135 (37.7) | | 144 (18-540) |
| Mind-body interventions | 19 (3.2) | NA | 4 (0.8) | 18 (18-18) | 5 (1.2) | 36 (18-54) | 2 (0.6) | | 144 (144-144) |
| Physical therapies | 23 (3.9) | 288 (288-432) | 16 (3) | 144 (54-288) | 10 (2.3) | 144 (144-1,439) | 12 (3.4) | | 288 (1-1,439) |
| Exercise | 102 (17.3) | 144 (18-1,439) | 91 (17.2) | 144 (18-1,439) | 93 (21.8) | 288 (18-2,158) | 77 (21.5) | | 288 (18-1,439) |
| **Total episode** | **588** |  | **528** |  | **427** |  | **358** | |  |

Abbreviations: CAM, Complementary and Alternative Medicine; MASTER, A Multicenter, Randomized, Controlled Trial for Assessing the Usefulness of Suppressing Thyroid Stimulating Hormone Target Levels after Thyroid Lobectomy in Low to Intermediate Risk Thyroid Cancer Patients

1. Multiple choice of cam modality was allowed.

2. The experience in CAM use were increased from the time of enrollment after thyroid cancer diagnosis to 12-month, regardless of CAM modalities. (p<0.001); but the costs paid among CAM users were not changed (p>0.1).

**Supplementary Table 4.** **Factors affecting CAM use or multiple use of 3 or more among CAM users in the MASTER trial (baseline, 3-month and 12-month follow-up^1^)**

|  | **CAM use vs non-use** | | | | | |
| --- | --- | --- | --- | --- | --- | --- |
|  | **Baseline** | | **3-month follow-up** | | **12-month follow-up** | |
|  | OR (95% CI) | *p*-value^2^ | OR (95% CI) | *p*-value^2^ | OR (95% CI) | *p*-value^2^ |
| **Total patients** |  |  |  |  |  |  |
| Low TSH | **1.28 (0.99-1.66)** | **0.06** | **1.29 (1.001-1.67)** | **0.05** | 1.13 (0.85-1.50) | 0.40 |
| Younger age (≤60 years) | 0.88 (0.64-1.22) | 0.44 | 1.09 (0.78-1.51) | 0.62 | **1.72 (1.15-2.57)** | **0.01** |
| Female | 1.34 (0.98-1.84) | 0.07 | 1.26 (0.92-1.73) | 0.15 | **1.47 (1.01-2.14)** | **0.04** |
| BMI ≥25 kg/m^2^ | 0.93 (0.72-1.21) | 0.60 | 0.84 (0.65-1.09) | 0.19 | 0.78 (0.58-1.04) | 0.09 |
| Current alcohol drinkers | **1.35 (1.03-1.78)** | **0.03** | 1.23 (0.94-1.62) | 0.14 | 0.89 (0.65-1.22) | 0.48 |
| LT4 use | **0.49 (0.38-0.64)** | **<0.001** | **0.57 (0.44-0.74)** | **<0.001** | **0.60 (0.44-0.80)** | **0.001** |
|  | **3+ multiple CAM use vs 1-2 CAM use** | | | | | |
| **Among CAM users** | OR (95% CI) | *p*-value^2^ | OR (95% CI) | *p*-value^2^ | OR (95% CI) | *p*-value^2^ |
| Low TSH | 1.26 (0.64-2.47) | 0.50 | 1.69 (0.65-4.42) | 0.29 | 0.52 (0.09-3.04) | 0.47 |
| Younger age (≤60 years) | **0.45 (0.21-0.95)** | **0.04** | 4.58 (0.59-35.22) | 0.14 | 0.60 (0.07-5.55) | 0.66 |
| Female | 1.44 (0.58-3.57) | 0.44 | 1.64 (0.43-6.26) | 0.47 | 0.77 (0.10-5.82) | 0.80 |
| BMI ≥25 kg/m^2^ | 0.74 (0.38-1.47) | 0.39 | 1.22 (0.48-3.09) | 0.67 | 0.96 (0.17-5.39) | 0.96 |
| Current alcohol drinkers | 1.05 (0.50-2.19) | 0.90 | 0.82 (0.30-2.25) | 0.70 | 3.95 (0.71-22.09) | 0.12 |
| LT4 use | **3.66 (1.88-7.14)** | **0.001** | 1.96 (0.78-4.91) | 0.15 | 2.79 (0.44-17.50) | 0.27 |

Abbreviations: CAM, Complementary and Alternative Medicine; MASTER, A Multicenter, Randomized, Controlled Trial for Assessing the Usefulness of Suppressing Thyroid Stimulating Hormone Target Levels after Thyroid Lobectomy in Low to Intermediate Risk Thyroid Cancer Patients; OR, odds ratio; CI, confidence interval; LR, logistic regression; TSH, thyroid-stimulating hormone; LT4, levothyroxine; BMI, body mass index.

1. The results from the 6-month follow-up survey were almost similar to the results from the 3-month follow-up survey and are therefore not presented.

2. p-value for the analysis of maximum likelihood estimates of parameters.

**Supplementary Table 5. Factors affecting CAM use or multiple use of 3 or more in the two randomly assigned arms (High-TSH group and Low-TSH group) of the MASTER trial (baseline, 3-month and 12-month follow-up^1^)**

|  | **CAM use vs non-use** | | | | | |
| --- | --- | --- | --- | --- | --- | --- |
|  | **After diagnosis (baseline)** | | **3-month follow-up** | | **12-month follow-up** | |
|  | OR (95% CI) | *p*-value^2^ | OR (95% CI) | *p*-value^2^ | OR (95% CI) | *p*-value^2^ |
| **High-TSH group** |  |  |  |  |  |  |
| Younger age (≤60 years) | **0.64 (0.42-0.99)** | **0.04** | 1.08 (0.68-1.70) | 0.75 | 1.69 (0.98-2.93) | 0.06 |
| Female | 1.24 (0.80-1.94) | 0.34 | 1.49 (0.94-2.35) | 0.09 | 1.42 (0.84-2.38) | 0.19 |
| BMI ≥25 kg/m^2^ | 1.02 (0.71-1.45) | 0.93 | 0.89 (0.62-1.27) | 0.52 | 0.88 (0.58-1.32) | 0.52 |
| Current alcohol drinkers | 1.27 (0.87-1.85) | 0.23 | 1.35 (0.92-1.98) | 0.12 | 0.83 (0.53-1.28) | 0.39 |
| LT4 use | **0.56 (0.37-0.83)** | **0.004** | **0.44 (0.29-0.67)** | **<0.001** | **0.37 (0.23-0.61)** | **<0.001** |
| **Low-TSH group** |  |  |  |  |  |  |
| Younger age (≤60 years) | 1.30 (0.79-2.15) | 0.30 | 1.12 (0.69-1.80) | 0.65 | **1.84 (1.01-3.35)** | **0.05** |
| Female | 1.46 (0.92-2.32) | 0.11 | 1.08 (0.69-1.68) | 0.75 | 1.53 (0.89-2.62) | 0.13 |
| BMI ≥25 kg/m^2^ | 0.86 (0.59-1.25) | 0.42 | 0.78 (0.54-1.13) | 0.19 | 0.67 (0.44-1.03) | 0.07 |
| Current alcohol drinkers | 1.44 (0.96-2.14) | 0.08 | 1.12 (0.76-1.66) | 0.58 | 0.97 (0.62-1.52) | 0.88 |
| LT4 use | **0.46 (0.32-0.65)** | **<0.001** | **0.67 (0.48-0.95)** | **0.02** | 0.83 (0.56-1.22) | 0.34 |
|  |  |  |  |  |  |  |
|  | **3+ multiple CAM use vs 1-2 CAM use** | | | | | |
|  | OR (95% CI) | *p*-value^2^ | OR (95% CI) | *p*-value^2^ | OR (95% CI) | *p*-value^2^ |
| **High-TSH group** |  |  |  |  |  |  |
| Younger age (≤60 years) | 0.47 (0.15-1.50) | 0.20 | 4.60 (0.60-35.32) | 0.14 | 0.35 (0.03-4.01) | 0.40 |
| Female | 3.72 (0.44-31.39) | 0.23 | 1.52 (0.40-5.79) | 0.54 | 0.81 (0.11-6.05) | 0.84 |
| BMI ≥25 kg/m^2^ | **0.29 (0.08-0.98)** | **0.05** | 0.69 (0.13-3.71) | 0.66 | 0.73 (0.07-7.63) | 0.79 |
| Current alcohol drinkers | 0.23 (0.05-1.08) | 0.06 | 0.61 (0.11-3.37) | 0.57 | 4.80 (0.47-48.60) | 0.18 |
| LT4 use | **4.17 (1.39-12.52)** | **0.01** | 0.44 (0.05-3.92) | 0.46 | 2.58 (0.19-35.29) | 0.48 |
| **Low-TSH group** |  |  |  |  |  |  |
| Younger age (≤60 years) | **0.34 (0.12-0.99)** | 0.05 | 2.89 (0.35-23.72) | 0.32 | 0.59 (0.06-5.45) | 0.64 |
| Female | 1.08 (0.36-3.24) | 0.89 | 1.13 (0.26-4.85) | 0.87 | 0.15 (0.01-2.87) | 0.20 |
| BMI ≥25 kg/m^2^ | 1.46 (0.61-3.47) | 0.40 | 1.67 (0.53-5.24) | 0.38 | 1.57 (0.09-26.06) | 0.75 |
| Current alcohol drinkers | 2.61 (0.98-6.95) | 0.05 | 0.98 (0.28-3.42) | 0.97 | 2.85 (0.19-42.15) | 0.45 |
| LT4 use | **4.42 (1.77-11.03)** | **0.002** | **3.58 (1.06-12.09)** | **0.04** | 2.60 (0.20-33.72) | 0.47 |

Abbreviations: CAM, Complementary and Alternative Medicine; MASTER, A Multicenter, Randomized, Controlled Trial for Assessing the Usefulness of Suppressing Thyroid Stimulating Hormone Target Levels after Thyroid Lobectomy in Low to Intermediate Risk Thyroid Cancer Patients; OR, odds ratio; CI, confidence interval; LR, logistic regression; TSH, thyroid-stimulating hormone; LT4, levothyroxine; BMI, body mass index.

1. The results from the 6-month follow-up survey were almost similar to the results from the 3-month follow-up survey and are therefore not presented.

2. p-value for the analysis of maximum likelihood estimates of parameters.

**Supplementary Table 6. Factors^1^ affecting CAM use or multiple use of 3 or more among CAM users in the MASTER trial**

|  |  | **Total patients** | | | | **CAM ever-users** | | | |
| --- | --- | --- | --- | --- | --- | --- | --- | --- | --- |
|  |  | **CAM**  **non-use** | **CAM**  **use** |  |  | **CAM**  **1-2 use** | **CAM**  **3+ use** |  |  |
|  | Reference | N (%) | N (%) | OR (95% CI) | *p*-value^2^ | N (%) | N (%) | OR (95% CI) | *p*-value^2^ |
| **All participants** |  |  |  |  |  |  |  |  |  |
| Low-TSH group^1^ | High-TSH group | 239 (48.8) | 345 (51.0) | **1.28 (1.00-1.64)** | **0.05** | 268 (49.7) | 77 (56.2) | 1.09 (0.73-1.61) | 0.69 |
| Younger age (≤60 years) | Age>60 years | 394 (80.4) | 565 (83.6) | 1.20 (0.88-1.64) | 0.25 | 450 (83.5) | 115 (83.9) | 0.98 (0.58-1.65) | 0.93 |
| Female | Male | 370 (75.5) | 533 (78.9) | 1.26 (0.93-1.70) | 0.13 | 421 (78.1) | 112 (81.8) | 1.24 (0.74-2.09) | 0.42 |
| BMI ≥25 kg/m^2^ | BMI<25 kg/m^2^ | 236 (48.2) | 275 (40.7) | **0.75 (0.59-0.96)** | **0.02** | 216 (40.1) | 59 (43.1) | 1.21 (0.81-1.80) | 0.36 |
| Current alcohol drinkers | Non-drinkers | 136 (27.8) | 226 (33.4) | **1.32 (1.01-1.73)** | **0.05** | 182 (33.8) | 44 (32.1) | 1.12 (0.73-1.73) | 0.60 |
| LT4 use | Non-use | 245 (50.0) | 246 (36.4) | **0.54 (0.42-0.69)** | **<0.001** | 174 (32.3) | 72 (52.6) | **2.29 (1.54-3.42)** | **<0.001** |
| **High-TSH group** |  |  |  |  |  |  |  |  |  |
| Younger age (≤60 years) | Age>60 years | 201 (80.1) | 269 (81.3) | 1.08 (0.71-1.65) | 0.72 | 218 (80.4) | 51 (85.0) | 1.32 (0.60-2.89) | 0.50 |
| Female | Male | 194 (77.3) | 257 (77.6) | 1.12 (0.74-1.70) | 0.61 | 206 (76.0) | 51 (85.0) | 1.50 (0.66-3.37) | 0.33 |
| BMI ≥25 kg/m^2^ | BMI<25 kg/m^2^ | 116 (46.2) | 145 (43.8) | 0.89 (0.63-1.25) | 0.50 | 122 (45.0) | 23 (38.3) | 0.83 (0.46-1.51) | 0.53 |
| Current alcohol drinkers | Non-drinkers | 78 (31.1) | 121 (36.6) | 1.24 (0.86-1.79) | 0.24 | 103 (38.0) | 18 (30.0) | 0.80 (0.42-1.51) | 0.49 |
| LT4 use | Non-use | 96 (38.3) | 82 (24.8) | **0.53 (0.37-0.76)** | **<0.001** | 61 (22.5) | 21 (35.0) | 1.74 (0.94-3.21) | 0.08 |
| **Low-TSH group** |  |  |  |  |  |  |  |  |  |
| Younger age (≤60 years) | Age>60 years | 193 (80.8) | 296 (85.8) | 1.39 (0.88-2.19) | 0.16 | 232 (86.6) | 64 (83.1) | 0.69 (0.33-1.44) | 0.32 |
| Female | Male | 176 (73.6) | 276 (80.0) | 1.40 (0.91-2.16) | 0.13 | 215 (80.2) | 61 (79.2) | 1.05 (0.52-2.11) | 0.90 |
| BMI ≥25 kg/m^2^ | BMI<25 kg/m^2^ | 120 (50.2) | 130 (37.7) | **0.63 (0.44-0.90)** | **0.01** | 94 (35.1) | 36 (46.8) | 1.71 (0.98-2.97) | 0.06 |
| Current alcohol drinkers | Non-drinkers | 58 (24.3) | 105 (30.4) | 1.40 (0.94-2.09) | 0.10 | 79 (29.5) | 26 (33.8) | 1.59 (0.88-2.89) | 0.13 |
| LT4 use | Non-use | 149 (62.3) | 164 (47.5) | **0.55 (0.39-0.78)** | **<0.001** | 113 (42.2) | 51 (66.2) | **2.97 (1.71-5.16)** | **<0.001** |

Abbreviations: CAM, Complementary and Alternative Medicine; MASTER, A Multicenter, Randomized, Controlled Trial for Assessing the Usefulness of Suppressing Thyroid Stimulating Hormone Target Levels after Thyroid Lobectomy in Low to Intermediate Risk Thyroid Cancer Patients; OR, odds ratio; CI, confidence interval; LR, logistic regression; TSH, thyroid-stimulating hormone; LT4, levothyroxine; BMI, body mass index.

1. The information of factors were measured or obtained at the time of baseline

2. p-value for the analysis of maximum likelihood estimates of parameters.

**Supplementary Figure 1. Flow diagram of study population.** CAM use was defined as having used CAM at least once within 12 months after a thyroid cancer diagnosis. Patients who responded "unknown" at all survey points were excluded. Among the remaining participants, those who reported no CAM use at all four survey points were classified as CAM non-users. Multiple selections of CAM modalities were allowed.

CAM experiences (%) by frequency of CAM use

|  | 1 | 2 | 3 |
| --- | --- | --- | --- |
| After diagnosis | 70.2 | 18.1 | 11.7 |
| 3-month | 73.7 | 20.7 | 5.6 |
| 6-month | 72.5 | 23.8 | 3.7 |
| 12-month | 71.4 | 25.9 | 2.6 |

**Supplementary Figure 2. CAM experiences by frequency of CAM use.** The frequency of CAM use at each survey time point (baseline, 3 months, 6 months, and 12 months post-lobectomy) is presented. At baseline, 70.2% of participants used one CAM modality, and this percentage remained relatively stable over time, with 73.7%, 72.5%, and 71.4% at 3, 6, and 12 months, respectively. The proportion of participants using two CAM modalities increased slightly over time, from 18.1% at baseline to 20.7% at 3 months, 23.8% at 6 months, and 25.9% at 12 months. Conversely, the proportion of participants using three or more CAM modalities decreased, from 11.7% at baseline to 5.6% at 3 months, 3.7% at 6 months, and 2.6% at 12 months. Multiple CAM modalities could be selected by each participant.

**Supplement material I: MASTER Trial: Detailed Study Protocol and Eligibility Criteria**

**1. Study Design and Overview**

The MASTER trial (Multicenter, Randomized, Controlled Trial for Assessing the Usefulness of Suppressing Thyroid Stimulating Hormone Target Levels after Thyroid Lobectomy in Low to Intermediate Risk Thyroid Cancer Patients) is a prospective, multicenter, randomized controlled trial designed to evaluate the effectiveness of thyroid stimulating hormone (TSH) suppression therapy in patients with papillary thyroid cancer (PTC) following lobectomy.

Patients were recruited from multiple centers across the country to ensure a diverse and representative sample. All participants provided written informed consent before enrollment. The study protocol was reviewed and approved by the Institutional Review Board (IRB) at each participating institution. The trial was registered at the Clinical Research Information Service (CRIS) on May 6, 2020 (trial registration number: KCT0004989).

**2. Inclusion Criteria**

Patients were eligible for the study if they met the following criteria:

- Age ≥ 19 years.
- Histologically confirmed papillary thyroid cancer (PTC).
- Clinical N0 or N1a disease.
- Primary tumor size ≤ 4 cm.
- Underwent lobectomy with complete tumor resection (R0).
- No evidence of contralateral thyroid malignancy.
- No history of previous thyroid surgery or radioactive iodine therapy.
- Provided written informed consent for study participation.

**3. Exclusion Criteria**

Patients were excluded from the study if they met any of the following criteria:

- Pathologic T4, N1b, or M1 disease.
- Aggressive histological variant of PTC (e.g., tall-cell, hobnail, columnar).
- Histological types other than PTC.
- Incomplete tumor resection (R1/R2).
- Candidates for completion thyroidectomy.
- Contralateral thyroid lobe nodules diagnosed as follicular neoplasm or malignancy on fine-needle aspiration.
- Active hyperthyroidism or pregnancy.
- Presence of significant comorbidities or conditions deemed unsuitable by the investigator.

**4. Randomization and Treatment Protocol**

Eligible patients were recruited before lobectomy and randomly assigned to either the **low-TSH group (target TSH 0.3–1.99 μIU/mL)** or the **high-TSH group (target TSH 2.0–7.99 μIU/mL)**. Levothyroxine (LT4) was administered as needed to maintain TSH within the target range. The TSH target levels were set based on the **2015 ATA guidelines**, which recommend suppression for high-risk patients and a weaker recommendation (TSH 0.5–2.0 μIU/mL) for lower-risk patients. The upper normal limit was set at 8.0 mIU/L based on Korean national survey data.

**5. Follow-Up Schedule**

After randomization, patients were followed according to the study protocol:

- **First 6 months**: Outpatient visits every 3 months.
- **Up to 2 years**: Outpatient visits every 6 months.
- **Thereafter**: Annual follow-up visits.

During each visit, patients underwent routine clinical assessments, laboratory tests (including TSH monitoring), and imaging studies as clinically indicated. Data on levothyroxine dosage, TSH levels, and patient adherence to treatment were collected throughout the study period.

**Supplemental material II. Complementary and Alternative Medicine (CAM): Survey for Thyroid Cancer patients (Initial & Follow-up)**

This survey concerns the **use of the Complementary and Alternative Medicine (CAM)** from the time after having diagnosis as thyroid cancer of yours. This survey excludes the regular treatment you receive at your medical institutes or hospitals as a thyroid cancer patient.

Please mark 'V' for the appropriate answer between 'Yes' and 'No'. And describe in details if you have any specific questions.

The information you provide will be used only for medical treatment and research purposes, and your personal information will be strictly protected in accordance with relevant laws.

Interview Date: _____ (Month) ______ (Day), 202___

| In the **past 3 months**, have you **paid for CAM use as thyroid cancer patients** at any institution other than the hospital you visited today?  * If you have been diagnosed with thyroid cancer within the past 3 months, please describe your experience from the time of diagnosis to the present. | | No | Yes | **If ‘Yes’, please answer** | |
| --- | --- | --- | --- | --- | --- |
|  |  |  |  | Total costs paid for CAM usage in the last 3 months* | Please provide details about the type of CAM you received. |
| 1) | **Pharmacotherapy prescribed by a physician in a hospital**  For example, use of injectable drugs including nutritional supplements or injectable preparations, etc | □ | □ | ______(KRW) | (___________________) |
| 2) | **Phytotherapy or nutritional supplementation in a pharmacy**  For example, Vitamins, iron, calcium, beverages, or traditional herbal formulations, etc | □ | □ | ______(KRW) | (___________________) |
| 3) | **Use of traditional oriental medication prescribed by an oriental medicine doctor**  For example, Herbal medicine, acupuncture, moxibustion, cupping, massage, or physical therapy, etc | □ | □ | ______(KRW) | (___________________) |
| 4) | **Other traditional oriental therapies**  For example, acupuncture, moxibustion or cupping, ginseng and deer antler velvet treatment, decoctions made from plant-based and animal-based medicinal herbs and ingredients | □ | □ | ______(KRW) | (___________________) |
| 5) | **Dietary nutritional products**  For example, whole grains/nuts/cereals, fermented liquids or fermented foods, dietary supplements, and health aids | □ | □ | ______(KRW) | (___________________) |
| 6) | **Mind-body interventions**  For example, prayer, laying on of hands, Buddhist rituals, Buddhist chants, traditional Korean shamanistic rituals, talismans, spiritual healing, meditation, hypnosis, Zen contemplation, breath control practices, energy flow practices, music therapy, art therapy, aromatherapy, and more) | □ | □ | ______(KRW) | (___________________) |
| 7) | **Physical therapies**  For example, hot springs, sauna, Korean herbal baths, steam treatments jade therapies, massage, acupressure, bodywork, or meridian therapies; chiropractic or physiotherapy; hydrotherapy, magnet therapy, jade, clay, germanium treatments, and more | □ | □ | ______(KRW) | (___________________) |
| 8) | **Exercise**  For example, fitness training, sports, yoga or Pilates, qigong, and related practices | □ | □ | ______(KRW) | (___________________) |

*KRW, Korean Won
